# Burden of aortic aneurysm and lead exposure risk factor in adults aged 60 years and older from 1990 to 2021: a global, regional, and national analysis

**DOI:** 10.3389/fpubh.2026.1696422

**Published:** 2026-04-10

**Authors:** Ze Song, Ming Qi, Xu Xie, Zhen Zhang, YuTong Xia, Guodong Wei, Song Wu, Lei Wang

**Affiliations:** Department of Vascular Surgery, First Affiliated Hospital of Dalian Medical University, Dalian, China

**Keywords:** aortic aneurysm, GBD (2021) database, global disease burden, lead exposure, old age

## Abstract

**Introduction:**

Aortic aneurysm (AA) is a life-threatening vascular disease that primarily affects older adults and represents a major global health challenge. However, the global burden of AA among the older adult population and the contribution of environmental lead exposure remain insufficiently characterized.

**Methods:**

Using data from the Global Burden of Disease (GBD) 2021 study, we analyzed the burden of AA among individuals aged ≥60 years. Secondary analyses were conducted for deaths, disability-adjusted life years (DALYs), the age-standardized death rate (ASDR), and the age-standardized DALYs rate at the global level, across five Socio-demographic Index (SDI) groups, 21 GBD regions, and 204 countries. The contribution of lead exposure to AA burden was assessed, and future trends in ASDR and age-standardized DALYs rate were projected for the next 15 years based on trends from 1990 to 2021.

**Results:**

Between 1990 and 2021, the global burden of AA among adults aged ≥60 years decreased modestly, although substantial regional heterogeneity persisted, particularly in less-developed regions. Lead exposure contributed disproportionately to the AA burden among older men. Projections based on historical trends indicate that both ASDR and the age-standardized DALYs rate will continue to decline steadily over the next 15 years, with a more pronounced decrease in males than in females.

**Discussion:**

Despite overall improvements, the burden of AA among older adults remains unevenly distributed worldwide. Given the notable contribution of lead exposure—especially among older men—region-specific strategies that integrate vascular risk management with environmental lead control are needed to mitigate the impact of population aging and address geographic and sex-related disparities in AA burden.

## Introduction

Aortic aneurysm (AA), characterized by abnormal segmental or diffuse dilation of the aorta exceeding 50% of its normal diameter, is most commonly observed as abdominal aortic aneurysm (1). Due to the insidious onset of AA and the high fatality of complications such as aneurysm rupture, the mortality rate of AA may be underestimated in real-world settings (2). It is estimated that once an AA ruptures, the overall mortality rate exceeds 80%, with even patients who receive timely medical attention facing a mortality rate of around 50%. This makes AA one of the most life-threatening cardiovascular diseases (3–5). Elizabeth M et al. conducted a prospective study on Abdominal aortic aneurysm(AAA) from 2003 to 2017 with follow-up of more than 3 years. The results showed that the three-year mortality rate for patients with large, untreated AAA exceeded 50% (6). The onset of AA is closely linked to age, as the aortic wall gradually loses its elasticity and becomes more fragile, leading to an increased incidence of aneurysm formation (7, 8). Studies have shown that the incidence of aortic aneurysm significantly increases, particularly in individuals aged 60 and above (9). With the accelerating global aging process, AA as a high-risk vascular disease, has become one of the major public health issues threatening the life and health of the older adults, particularly in aging countries such as China and Japan (10, 11). Therefore, under the background of an aging population, conducting a comprehensive study of the epidemiological characteristics of AA across different countries and regions, identifying high-risk factors, and developing targeted prevention and intervention strategies based on regional characteristics is of significant importance in alleviating the global disease burden of AA.

The Global Burden of Disease (GBD) provides comprehensive and systematic epidemiological data on various diseases, injuries, and health risk factors at the global, regional, and national levels, allowing for a multidimensional understanding of disease burden distribution characteristics and trends (12–14). With the changes in global disease burden and health risks, the update of the GBD 2021 database provides reliable data support for research in fields such as epidemiology and public health (15). A previous study by Wang et al. (10), based on GBD 2019, found that although the burden of AA has decreased in some developed regions, the global number of AA-related deaths continues to rise. Furthermore, this trend exhibits unequal effects across different countries and regions. In addition, studies have shown that various factors commonly found in the older adults, such as hypertension, hyperlipidemia, and long-term smoking, further exacerbate the risk of developing AA (16, 17). AA is often overlooked in cardiovascular diseases, particularly in the older adults, where it is frequently underdiagnosed. However, AA has gradually become a serious health threat in the older adult population, comparable to cancer in terms of its severity (18). It is worth noting that epidemiological analyses of the disease burden and risk factors of AA in older adults at the global, regional, and national levels are still lacking.

AA remains a leading cause of death among older adults worldwide. Its pathogenesis involves degenerative changes of the vascular wall, inflammation, and oxidative stress, and is strongly influenced by environmental risk factors. Among environmental exposures, air pollution and heavy metals have drawn increasing attention; notably, lead exposure. Epidemiological evidence indicates that even “low-level” lead is associated with higher cardiovascular mortality, providing biological plausibility and population-level support for an environmental etiology of AA (19). Mechanistic and population studies further corroborate this link: lead can induce oxidative stress, reduce nitric oxide bioavailability, activate sympathetic and endothelin pathways, and disrupt Ca^2+^ signaling in vascular smooth muscle and extracellular matrix metabolism, thereby promoting arterial stiffness and hypertension and accelerating degeneration of the vessel wall (20). Occupational studies also suggest that long-term lead exposure is associated with impaired aortic elastic properties, indicating adverse effects on the mechanics of proximal large arteries (21). Within environmental pollution, lead exposure is a salient GBD-identified risk factor for AA burden; the GBD 2021 comparative risk assessment attributes a portion of AA deaths and DALYs to lead exposure, motivating its inclusion as a primary environmental exposure in AA research (22). In sum, among environmentally attributable factors for AA, lead exposure is an intervenable and regulatable risk factor particularly relevant to older adults, necessitating burden assessments and prevention strategies to inform precision prevention in aging populations.

In recent years, significant progress has been made in the research of AA, particularly in areas such as early diagnosis, pathological mechanisms, treatment strategies, and risk assessment. Notably, the integration of CT and MRI imaging for aneurysm modeling not only allows for early risk assessment of AA but also provides crucial support for surgery, thereby improving overall repair success rates (23–25). Overall, personalized and precision medicine will play an increasingly pivotal role in the prevention and treatment of AA. In the context of an aging population, conducting comprehensive studies on the epidemiological characteristics of AA across different countries and regions, identifying high-risk factors, and developing targeted prevention and intervention strategies based on regional characteristics will be crucial in reducing the incidence and mortality of the disease.

Our study aims to conduct a multidimensional analysis of the mortality rate and DALYs for AA using the latest GBD 2021 database. The goal is to explore the differences in the disease burden of AA across different countries and regions, identify related risk factors, and provide more precise information for public health policies and disease prevention strategies. By revealing the current state and challenges of aortic aneurysm on global health, the study seeks to effectively reduce the impact of AA on the global disease burden.

## Methods

### Overview

Overall, this study conducted a secondary data analysis based on the GBD 2021 database to explore the latest epidemiological characteristics of AA in individuals aged 60 and above. The key indicators analyzed in this study include AA mortality rates and DALYs, with a time span covering 1990 to 2021. DALYs combine Years of Life Lost (YLL) due to premature death and Years Lived with Disability (YLD) to assess health loss, representing the total number of health years lost due to both death and disability caused by a disease. In the GBD framework, DALYs equal YLLs plus YLDs. Because the GBD outputs for AA show identical DALYs and YLLs, YLDs are effectively zero, indicating that the burden is driven almost entirely by premature mortality. Accordingly, we selected DALYs as the primary outcome to capture overall health loss and to assess temporal trends and regional heterogeneity from 1990 to 2021, ensuring comparability across regions and over time. This metric has become an essential measure in public health. In our study, the AA population aged 60 and above was categorized into eight age groups with 5-year intervals. Additionally, we reported the ASDR and age-standardized DALYs rate per 100,000 people to adjust for the impact of demographic differences. The study covers global data, 21 regions, and 204 countries, and includes a stratified analysis by the Socio-Demographic Index (SDI) to examine the relationship between socioeconomic development and AA burden. The SDI is a composite indicator that measures the socioeconomic development level of countries and regions based on per capita income, average years of schooling, and fertility rates for women under 25. SDI values range from 0 to 1, with higher values indicating higher levels of socioeconomic development. In addition, the study investigates the association between certain AA risk factors and regional differences.

### Data sources and definition

The GBD database is a global epidemiological research project led by the Institute for Health Metrics and Evaluation (IHME) at the University of Washington, aimed at measuring the health loss caused by diseases, injuries, and risk factors globally and across regions. The data for GBD 2021 includes, but is not limited to, mortality rates and census data provided by national governments, multi-country studies and health surveys from the World Health Organization (WHO), official reports from healthcare organizations, agencies, or government bodies, as well as published scientific literature. GBD 2021 includes 371 diseases and injuries, covering 95 infectious diseases, 234 non-communicable diseases, 40 types of injuries, as well as maternal and neonatal-related diseases. The GBD assessments cover key indicators such as incidence, mortality, quality of life, as well as considering DALYs and YLL due to diseases. The GBD database provides estimated values for all indicators, with a 95% uncertainty interval (95% UI) to indicate the credible range of the estimate for each indicator. We collected data from the Global Health Data Exchange (GHDx) results tool, with all data downloaded from the GBD 2021 database^1^. This study adhered to the guidelines for accurate and transparent health estimates reporting (GATHER) (26).

AA in GBD 2021 is defined as an abnormal enlargement or weakening of the aorta caused by atherosclerosis, hypertension, cellular or extracellular structural changes, or inflammation, affecting both the abdominal and thoracic aorta and potentially leading to rupture. However, the incidence, prevalence, and YLDs of AA have not been included in GBD 2021 statistics.

### Statistical analysis

In our study, we first calculated the estimated annual percentage change (EAPC) in the numbers of deaths and DALYs across age strata among adults aged 60 years and older worldwide between 1990 and 2021. The EAPC values were used to describe the trends of changes in the time series data. The EAPC values were calculated using a linear regression model to fit the time series data, and the formula is as follows, where *β* represents the regression coefficient. A positive EAPC value indicates an increasing trend. Additionally, we calculated the 95% confidence interval (CI) for the EAPC values to assess their reliability.


$$EAPC=\left(e^{\beta }-1\right)\times 100$$

To further identify the underlying causes of the changes in AA burden, we employed the decomposition analysis method proposed by Das Gupta. This method allows us to quantify the relative contributions of population size, age structure, and epidemiological changes to the mortality and DALYs of AA patients aged 60 and above (27).

We used frontier analysis to approximate the minimum empirically achievable AA burden at a given level of development. Specifically, using all country–year observations (1990–2021), we fit a robust LOESS curve of SDI versus age-standardized DALY rate and took the lower empirical envelope as the frontier; this is an observational LOESS boundary conditional on SDI, not a quality benchmark. To quantify each country’s gap to best-observed performance, we defined the effective difference as:


$$Effectivedifference_{i,t}=max\;\left\{0,ASDR_{i,t}-ASDR_{frontier}\;\left(SDI_{i,t}\right)\right\}$$

Where ASDR is the age-standardized DALYs rate and ASDR_frontier_ is the LOESS frontier evaluated at the same SDI. We used bootstrap resampling (100 replicate samples of country–years) to characterize uncertainty in the frontier and in effective differences.

This study applies data on AA mortality and DALYs for males and females from 1990 to 2021 and uses an Autoregressive Integrated Moving Average (ARIMA) model to predict the disease burden over the next 15 years. ARIMA is a widely used statistical model for time series forecasting, combining three components: autoregression (AR), differencing (I), and moving average (MA). The formula is as follows:


$$Y_{t}=\phi_{1}Y_{t-1}+\phi_{2}Y_{t-2}+\cdots +\phi_{p}Y_{t-p}+\vartheta_{1}\in_{t-1}+\vartheta_{2}\in_{t-2}+\cdots +\vartheta_{q}\in_{t-q}+\in_{t}$$

In this study, we selected the appropriate AR order (*p*) and MA order (*q*) by observing the Autocorrelation Function (ACF) and Partial Autocorrelation Function (PACF). We then fitted the ARIMA model using the training data, estimated the parameters *ϕ_i_* and *θ_i_*, and ultimately used the fitted ARIMA model to predict future data.

All data processing, data analysis, and result visualization in this study were performed using R language (version 4.2.2).

## Results

### The deaths and DALYs of AA between 1990 and 2021

**Figure 1:**
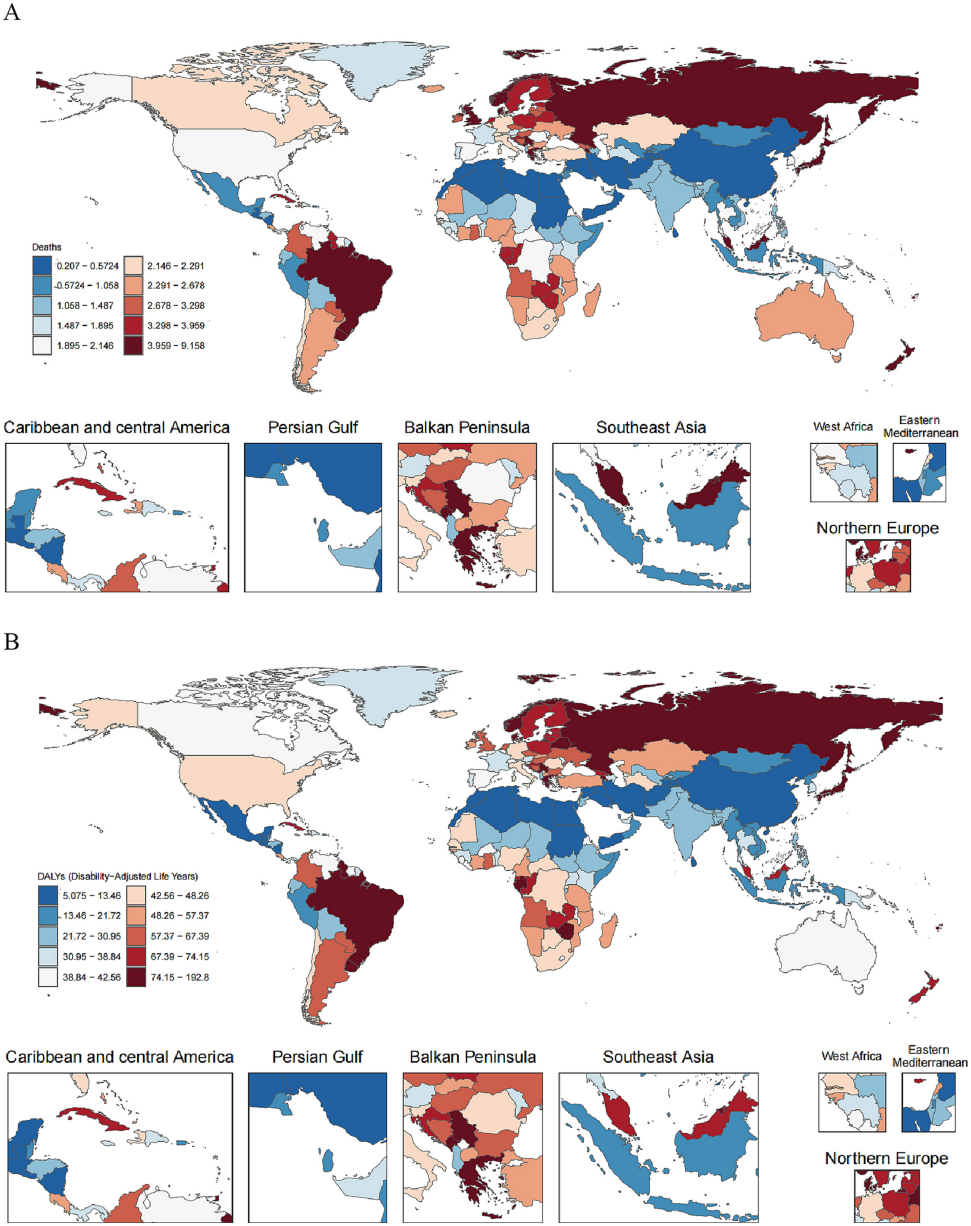
The world map of the global burden distribution of age-standardized mortality rate and age-standardized DALYs rate for aortic aneurysm: **(A)** age-standardized mortality rate; **(B)** age-standardized DALYs rate.

Globally, AA mortality and disability burden exhibited notable age-specific trends from 1990 to 2021. Among older adults (≥60 years), absolute AA death and DALYs counts increased substantially in every age group, reflecting population growth and aging. However, nearly all age-specific AA mortality and DALYs rates declined over this period, indicating improved outcomes; these declines were generally larger in the younger older adult cohorts and diminished with advancing age. For example, in the 75–79 year group, the number of AA deaths rose from approximately 16,500 (95% UI: 15,500–17,700) in 1990 to 20,700 (18,700–23,000) in 2021, even as the mortality rate nearly halved from 26.7 (25.0–28.6) to 15.7 (14.1–17.4) per 100,000 (EAPC −2.03% per year, 95% UI: −2.18 to −1.89). Similarly, the AA DALYs rate in this age group dropped markedly (from 425 to 249 per 100,000; EAPC –2.04%) despite total DALYs increasing from about 263.1 thousand (246.7–281.9) to 330.0 thousand (297.1–365.9). By contrast, much smaller rate reductions were observed at the highest ages – for instance, the mortality rate at 90–94 years declined only modestly (from 71.9 to 65.4 per 100,000; EAPC −0.26%), and the ≥95-year group even experienced a slight increase in AA mortality and DALYs rates over time (e.g., mortality rising from 84.4 to 90.9 per 100,000; EAPC +0.43%) (Table 1). Figure 1 illustrates the AA disease burden across the 204 countries in 2021.

**Table 1:** Counts and rates of aortic aneurysm deaths and DALYs in 1990 and 2021, and temporal trends during 1990–2021.

	Deaths					DALYs				
Age	Number of cases, 1990	Rates of cases, 1990	Number of cases, 2021	Rates of cases, 2021	EAPC, 1990-2021	Number of cases, 1990	Rates of cases,1990	Number of cases, 2021	Rates of cases, 2021	EAPC, 1990-2021
Global										
60-64 years	8079.211 (7412.985–9084.981)	5.022 (4.608–5.647)	12539.139 (11250.318–14263.715)	3.897 (3.496–4.433)	–1.156 (–1.275––1.037)	233041.611 (213791.422–262092.524)	144.851 (132.885–162.908)	361738.12 (324535.95–11535.799)	112.419 (100.857–127.894)	–1.152 (–1.269–1.034)
65-69 years	11687.919 (10915.355–12786.63)	9.439 (8.815–10.326)	17086.619 (15507.137–19296.907)	6.142 (5.574–6.936)	–1.696 (–1.836–1.555)	284170.929 (265349.948–310924.78)	229.483 (214.284–251.088)	415256.083 (376788.582–469037.541)	149.267 (135.44–168.599)	–1.692 (–1.832–1.553)
70-74 years	13412.449 (12565.371–14487.39)	15.855 (14.854–17.126)	20334.98 (18523.548–22485.372)	9.845 (8.968–10.886)	–2.053 (–2.207–1.898)	268037.621 (251071.396–289605.921)	316.851 (296.795–342.347)	406025.145 (369882.307–449134.914)	196.565 (179.067–217.435)	–2.056 (–2.213–1.899)
75-79 years	16503.346 (15481.724–17684.182)	26.667 (25.016–28.575)	20735.145 (18672.196–22976.951)	15.671 (14.112–17.365)	–2.033 (–2.176–1.89)	263052.371 (246738.668–281915.269)	425.049 (398.689–455.528)	330047.83 (297054.476–365965.848)	249.434 (224.499–276.579)	–2.041 (–2.188–1.895)
80-84 years	13637.42 (12521.957–14724.574)	38.174 (35.052–41.217)	21888.482 (18948.844–24087.691)	24.407 (21.129–26.86)	–1.711 (–1.849–1.572)	170500.026 (156550.035–184094.081)	477.269 (438.22–515.322)	272344.843 (235738.466–299808.512)	303.686 (262.867–334.31)	–1.725 (–1.863–1.586)
85-89 years	8117.769 (7217.375–8909.115)	52.504 (46.681–57.623)	19241.669 (15741.034–21723.504)	39.935 (32.67–45.086)	–0.939 (–1.093–0.784)	80564.456 (71626.429–88444.101)	521.078 (463.268–572.042)	189833.194 (155355.922–214303.257)	393.987 (322.431–444.773)	–0.957 (–1.109–0.804)
90-94 years	3198.96 (2709.767–3590.741)	71.856 (60.867–80.656)	12239.832 (9756.638–13976.882)	65.371 (52.109–74.649)	–0.256 (–0.346–0.166)	27576.183 (23359.6–30953.486)	619.423 (524.709–695.284)	105409.596 (84029.039–120363.393)	562.977 (448.787–642.844)	–0.259 (–0.349–0.17)
95+ years	911.604 (723.3–1058.794)	84.443 (67.001–98.078)	5113.609 (3637.241–6015.364)	90.919 (64.67–106.952)	0.427 (0.337–0.516)	7430.558 (5897.157–8628.39)	688.306 (546.264–799.263)	41414.492 (29485.063–48693.072)	736.345 (524.241–865.757)	0.389 (0.3–0.478)

### Attributed to lead exposure risk factor contribution to AA burden

**Figure 2:**
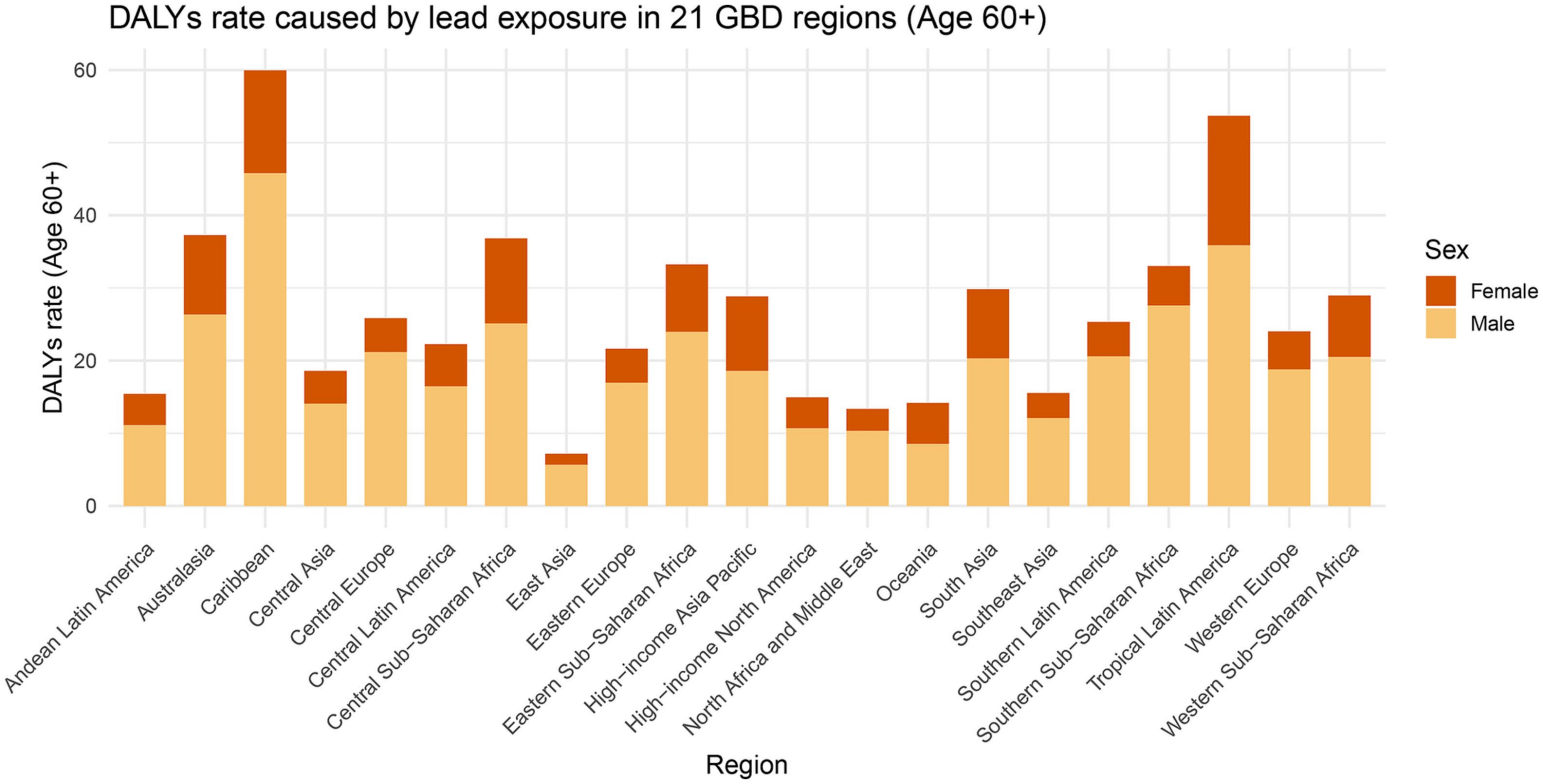
Variation in the incidence of aortic aneurysm DALYs attributable to lead exposure (per 100,000 population) across the 21 GBD regions, and gender differences in each region.

Across the 21 GBD regions, AA burden attributable to lead exposure shows marked geographic heterogeneity. By DALYs rate, the Caribbean and Tropical Latin America rank highest, followed by High-income Asia Pacific, South Asia, and Western Sub-Saharan Africa; East Asia and Andean Latin America are lowest (Figure 2). In most regions, men bear a substantially higher burden than women, with male contributions predominating; the sex gap is relatively smaller in the Caribbean and Tropical Latin America. The mortality pattern broadly mirrors the DALYs distribution, but South Asia shows the highest death rate and the most pronounced disparity; the Caribbean, Southeast Asia, and Tropical Latin America are also elevated, whereas East Asia, Australasia, and Andean Latin America remain low (Supplementary Figure S1).

### Global burden inequality by age and gender

**Figure 3:**
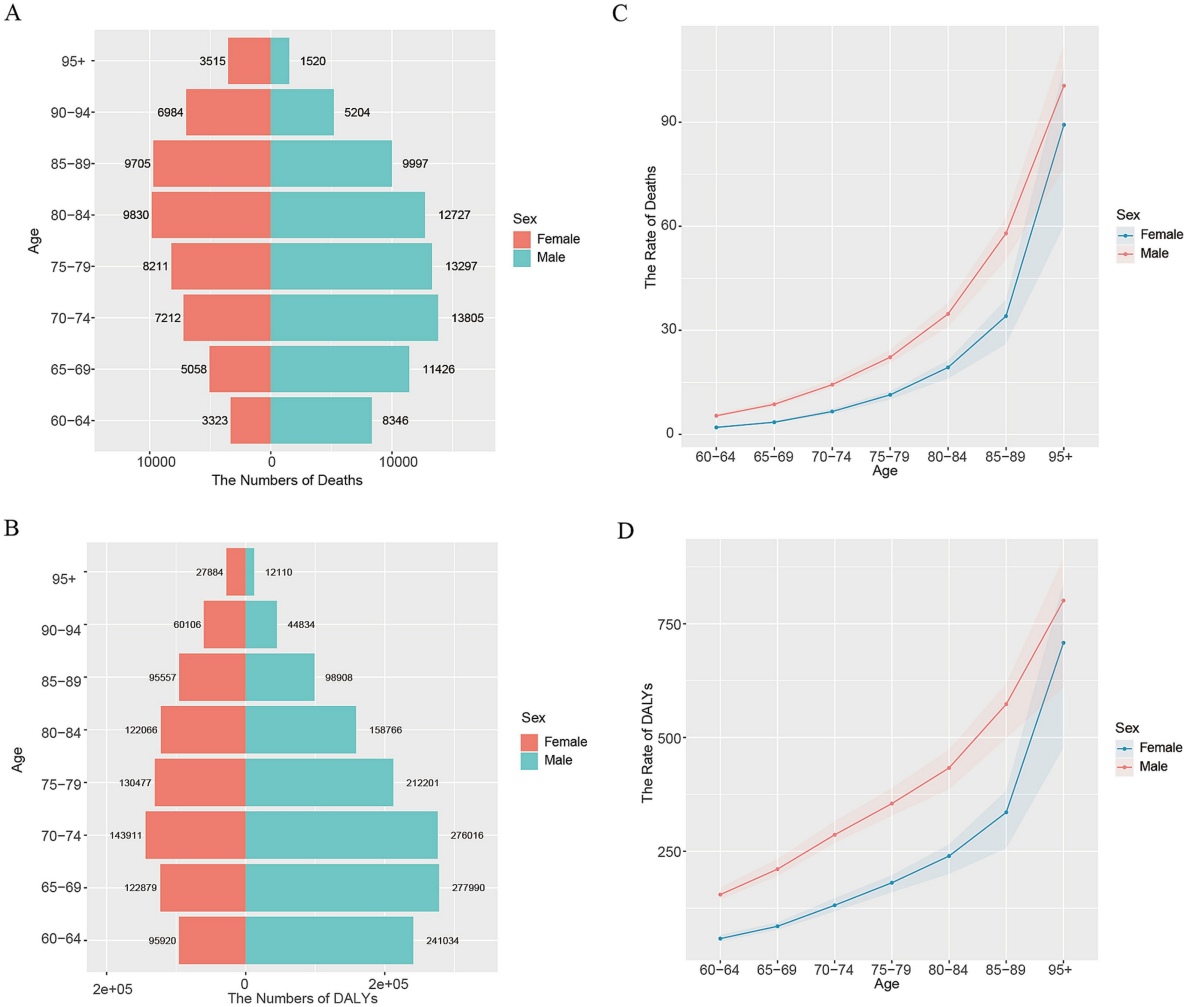
The age and sex distribution pattern chart of global death cases and DALYs for aortic aneurysm over 60 years old in 2021. **(A)** The distribution of death cases for aortic aneurysm over 60 years old in 2021; **(B)** the distribution of DALYs for aortic aneurysm over 60 years old in 2021; **(C)** the age-related changes in global mortality rates for aortic aneurysm over 60 years old in 2021, by gender; **(D)** the age-related changes in DALYs rates for global aortic aneurysm over 60 years old in 2021, by gender.

To better understand the disease burden of AA in individuals aged 60 and above in 2021, we analyzed and presented the age and gender distribution of mortality and DALYs among this population. The results indicated that across five-year age intervals from 60 to 85, the number of AA-related deaths increased with age, with males experiencing higher mortality than females. Notably, for those aged 85 and above, AA-related mortality decreased with age, with a higher number of female deaths (Figure 3A). The DALYs distribution showed that, among male patients, DALYs peaked between the ages of 65 and 75, gradually declining in those aged 75 and above. The overall trend for female patients was similar to that of males(Figure 3B). We further illustrated the mortality and DALYs rates for AA in 2021 with line graphs (Figures 3C,D). These results showed that as age increased, the mortality and DALYs rates for AA patients over 60 gradually increased, with male patients bearing a heavier disease burden.

### Decomposition analysis

**Figure 4:**
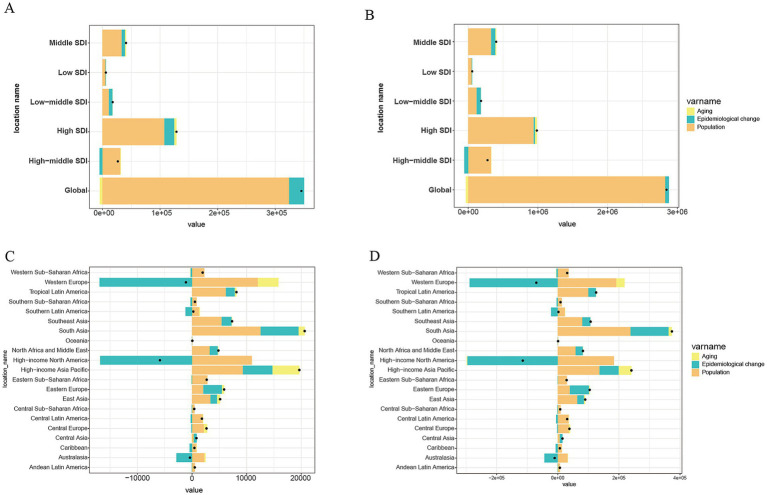
The decomposition analysis stacked bars display the contributions of three components to the change from 1990 to 2021: population growth, population aging, and epidemiological change. Black dots indicate the overall net change. **(A)** Mortality rate globally and in 5 SDI regions; **(B)** DALYs rate globally and in 5 SDI regions; **(C)** Mortality rate in 21 GBD regions; **(D)** DALYs rate in 21 GBD regions.

We employed decomposition analysis to further explore the impact of population growth, aging, and epidemiological trends on the mortality and DALYs of AA in individuals aged 60 and above. The results indicated that population growth was the primary driver of changes in the AA burden across global and the five SDI regions, followed by the impact of epidemiological trends (Figures 4A,B). Compared to the other four SDI regions, high SDI regions were most affected by population growth in terms of both mortality and DALYs. Based on the decomposition analysis across 21 regions, the epidemiological trend had a significant impact in regions such as Western Europe and High-income North America, while population factors were particularly influential in regions like Western Europe, South Asia, and High-income North America. Population aging had a notable effect in Western Europe and the High-income Asia Pacific region (Figures 4C,D).

### Relationship between SDI and AA burden

**Figure 5:**
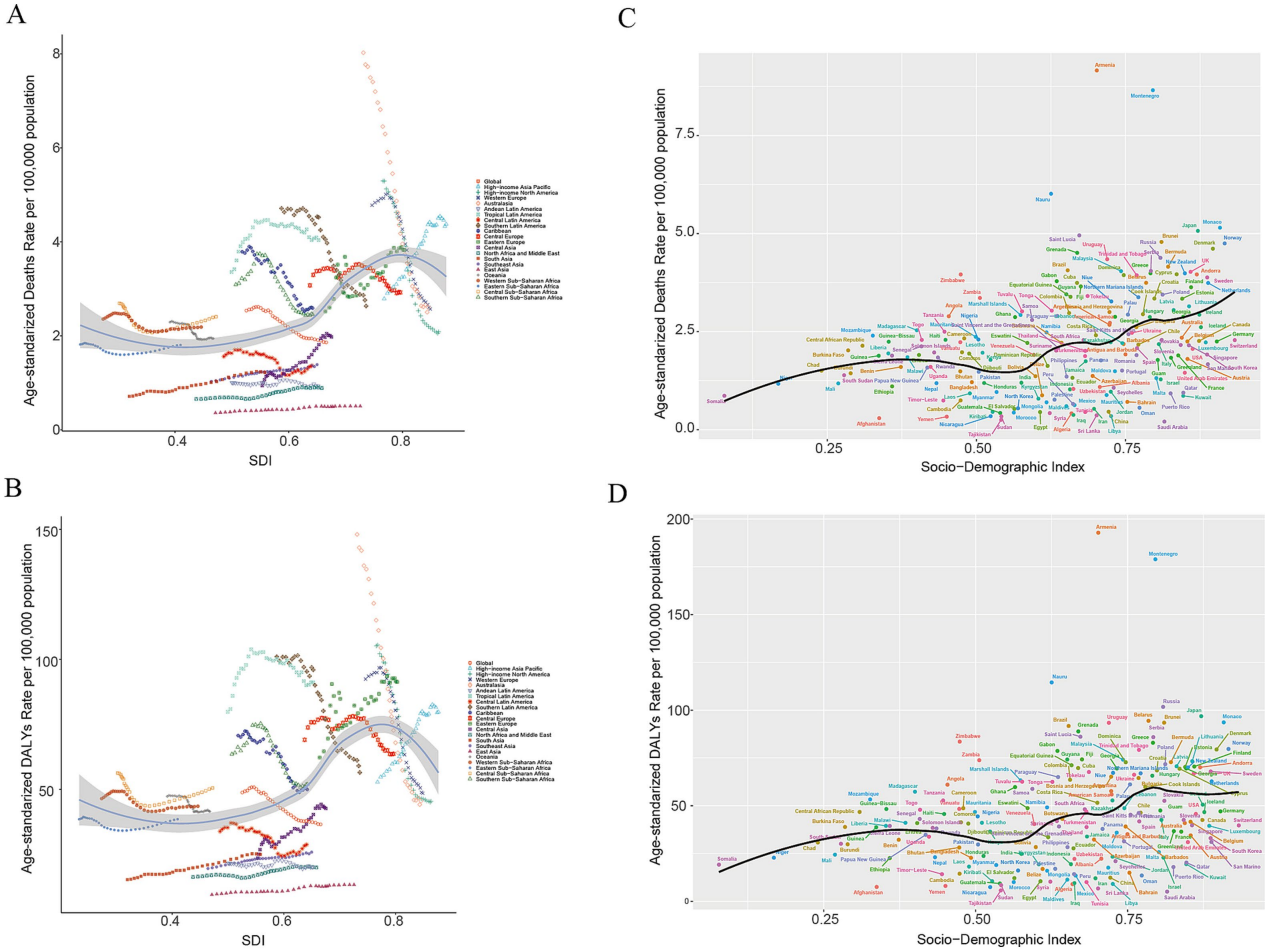
Association between the SDI and the age-standardized mortality rate and age-standardized DALYs rate of aortic aneurysm across 21 GBD regions and 204 countries, 1990–2021. **(A)** SDI values and changes in the age-standardized mortality rate of aortic aneurysm in 21 GBD regions; **(B)** SDI values and changes in the age-standardized DALYs rate of aortic aneurysm in 21 GBD regions; **(C)** SDI values and changes in the age-standardized mortality rate of aortic aneurysm in 204 countries; **(D)** SDI values and changes in the age-standardized DALYs rate of aortic aneurysmin 204 countries.

To further examine the association between SDI and AA burden, we assessed correlations at both the regional (21 GBD regions) and national (204 countries) levels. Figures 5A,B depict the relationship between SDI and age-standardized mortality and DALYs rates across regions from 1990 to 2021; higher-SDI regions (e.g., Australasia, High-income Asia Pacific, High-income North America) showed greater temporal fluctuations than lower-SDI regions. At the country level (Figures 5C,D), nonparametric correlations indicated a modest monotonic association between SDI and the outcomes (Spearman’s *ρ* for mortality = 0.487; Spearman’s ρ for DALYs = 0.466; both *p* < 0.001), suggesting that SDI is related to variation in age-standardized AA mortality and DALYs rates.

**Figure 6:**
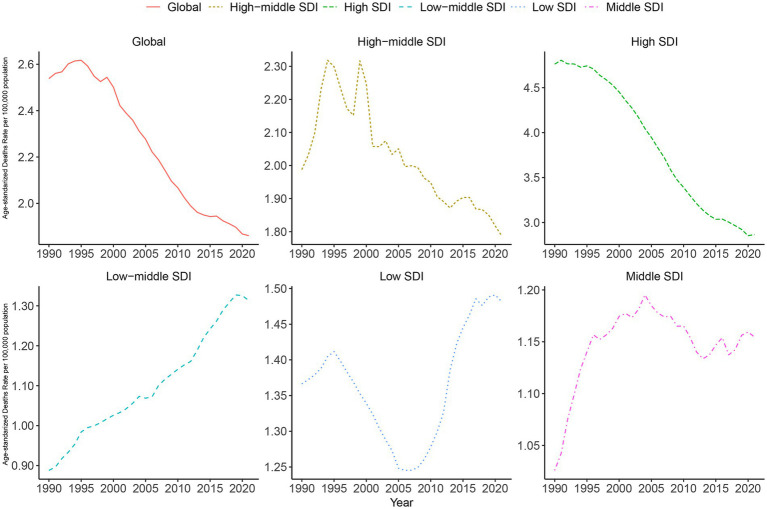
The trends in age-standardized mortality rate globally and in 5 SDI regions from 1990 to 2021.

Next, we present the trends in age-standardized mortality rates (Figure 6) and DALYs rates (Supplementary Figure S2) for AA across the globe and the five SDI regions. The results indicate a decline in the global age-standardized mortality rate for AA from 1990 to 2021. In contrast, regions with medium and low-middle SDI exhibited an increase in age-standardized mortality rates. Notably, low SDI regions saw a decrease in age-standardized mortality rates from 1990 to 2005, followed by an increase from 2005 to 2021.

### Frontier analysis

**Figure 7:**
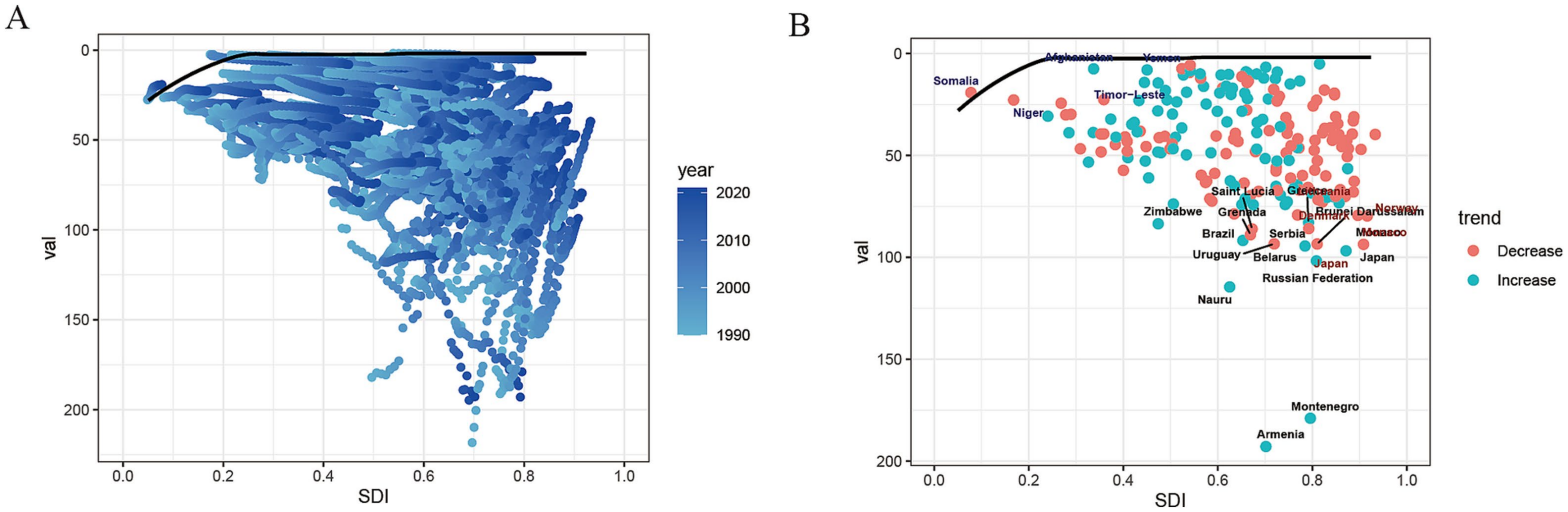
Frontier analysis of age-standardized DALYs rate for aortic aneurysm. The black line represents the optimal aortic aneurysm control situation for the SDI index. **(A)** Frontier analysis based on the SDI and aortic aneurysm DALYs rate from 1990 to 2021 in 204 countries; **(B)** Frontier analysis based on the SDI and aortic aneurysm DALYs rate in 204 countries in 2021. The five countries with the largest effective differences from the frontier are marked in red font, and the five countries with the smallest effective differences are marked in blue font.

To investigate the current disease control practices for AA across different countries, we conducted a frontier analysis to define the DALYs rates of the best-performing countries or regions as the boundary. The DALYs rates of each country are represented by blue dots, and the distance from the point to the boundary is defined as the “effective difference,” which represents the gap between the best possible disease control practices achievable at the country’s current level of social development and the actual situation. The results show that countries with smaller effective differences have smaller fluctuations in DALYs rates, while those with larger effective differences show greater variations in DALYs rates over time (Figure 7A). Notably, some high SDI countries, such as Japan, Norway, and Armenia, exhibit higher effective differences. In contrast, lower SDI countries, including Somalia, Afghanistan, and Yemen, have DALYs rates closer to the boundary, suggesting that these regions have relatively ideal AA disease prevention and control practices (Figure 7B).

### ARIMA forecasting model

To forecast the future global burden of AA, we utilized age-standardized mortality rates and DALYs rates from 1990 to 2021 to predict the trend over the next 15 years. The results indicate a steady decline in both the age-standardized mortality rate and DALYs rate for AA in the coming years. Notably, the decline is more pronounced in males compared to females (Supplementary Figure S3).

## Discussion

Aging is an unstoppable trend in global social development. AA poses a significant challenge to public health and is currently one of the leading disease burdens globally. Based on GBD 2021 data, this study conducted a multi-dimensional analysis of the mortality rates, DALYs, and age-standardized rates of AA among individuals aged 60 and above. The findings revealed significant regional and national differences in the epidemiological characteristics of AA, highlighting the necessity of developing region-specific and personalized public health intervention strategies. In this study, we also analyzed the relative contribution of various attributable risk factors to the DALYs rates of AA across different regions, helping identify high-risk populations and informing targeted public health policies and health interventions. Our analysis showed a declining trend in the global age-standardized mortality and DALYs rates for AA between 1990 and 2021. Predictions for the next 15 years suggest that this downward trend will continue, likely reflecting advances in AA treatment and diagnosis, as well as socioeconomic development. In the decomposition analysis of AA disease burden, we identified population factors and disease prevalence trends as key drivers. These factors are closely linked to the global aging population. Changes in population structure have led to an increase in high-risk groups for AA, while the aging process has exacerbated the prevalence of chronic diseases such as hypertension and arteriosclerosis, both of which are strongly associated with the occurrence of AA. Compared to previous studies, our focus on the global and regional epidemiological characteristics of AA in older adult populations, based on the GBD 2021 update, aims to guide the rational allocation of medical resources and the optimization of public health policies (10, 28–30).

The global burden of AA related to lead exposure shows significant regional inequalities and gender differences. Lead exposure is considered one of the attributable risk factors for AA. As the human body is unable to effectively metabolize lead, prolonged exposure leads to its accumulation in the body. With increasing age, the risk of diseases associated with lead exposure rises. This study focuses on high-risk populations aged 60 and above, indirectly reflecting the disease burden caused by long-term lead exposure in different regions (31). The observed geographic heterogeneity in the burden of AA in older populations due to lead exposure highlights the long-term impact of environmental, socioeconomic, and policy factors on global health outcomes. Regions such as the Caribbean and Tropical Latin America bear the highest DALYs rates, likely related to elevated levels of lead exposure. These exposures stem from sources such as leaded gasoline, industrial emissions, battery recycling, and lead-glazed ceramics, which are widespread in both urban and rural environments (32). These disparities highlight the need for tailored, multi-sectoral interventions, strengthening monitoring of lead content in the environment and production, to equitably reduce the burden of AA (33). To address AA directly, pair lead control with targeted vascular screening where recommended. For AA policy, we recommend a dual approach of lead abatement plus targeted vascular screening: offer abdominal aortic ultrasound screening to men aged ≥60 years and to workers with a history of occupational lead exposure.

Our study reveals differences in the age and gender distribution of AA disease burden in individuals aged 60 and above. Female patients show higher AA mortality rates and DALYs, with the peak age for AA-related deaths occurring later in women compared to men. Current research consistently indicates that the prevalence of AA is significantly higher in men than in women (34, 35), but female patients have a higher risk of AA rupture and generally worse prognosis (36). Moreover, this age distribution difference between genders has attracted the attention of scholars. Studies have shown that the prevalence of AAA in older adult women increases rapidly with age. Compared to individuals under 70, the prevalence in the 71–80 age group is 2.7 times higher, and in those over 81, it is as high as 7.3 times (37). The differential distribution of AA across genders may stem from several factors. One key explanation is that premenopausal women benefit from higher estrogen levels, which offer vascular protection. Estrogen helps maintain the elasticity of blood vessel walls and reduces the risk of arteriosclerosis, which may delay the onset of AA in women. As a result, the peak incidence and mortality of AA in women occur later than in men, typically after menopause, when estrogen levels decline (38, 39). Moreover, studies suggest that the gender differences in AA may be linked to smoking, which is a significant risk factor for the disease. Smoking can affect hormone levels and further negatively impact the structure and function of blood vessel walls, potentially exacerbating the development and progression of AA, particularly in women (40). Additionally, studies have shown that as age increases, the decline in smoking rates among women aged 65 and above is slower compared to men. This slower reduction in smoking prevalence among older women may contribute to their increased risk of AA and its associated complications (41, 42), and the incidence of AAA is higher among female smokers (43). Overall, the gender differences in AA among the older adult suggest the need for gender-specific prevention, screening, and intervention strategies to effectively reduce the disease burden and improve health outcomes in the older adult population.

Integrating our results, the AA burden attributable to lead exposure among adults aged 60 and older shows pronounced regional and sex heterogeneity, with men more heavily affected—underscoring the need to treat lead as an intervenable environmental factor in prevention and control strategies. An occupational study further suggests that long-term lead exposure can impair aortic elastic parameters before overt hypertension develops, implying earlier and more insidious adverse effects on the mechanics of proximal large arteries (21). Evidence also indicates that related metal toxicants such as cadmium are independently associated with higher risk of abdominal aortic aneurysm, supporting a “metal exposure–vascular remodeling–aneurysm” toxicological pathway (44). Accordingly, in high-burden regions and populations—especially older men—strengthening drinking-water and soil remediation, replacing aging pipe networks and controlling workplace lead exposure, together with rigorous blood-pressure control and smoking mitigation, may yield substantial secondary and tertiary prevention benefits for AA.

The correlation analysis between SDI and the disease burden of AA shows that regions with higher SDI have a general downward trend in AA mortality and DALYs. High-SDI regions typically have more advanced medical technologies and equipment, and the residents in these areas generally have higher health awareness, leading them to proactively engage in health screenings. Studies have shown that post-repair mortality rates of AA are associated with socioeconomic status (45). Moreover, the differences in patient demographics across regions are also important factors influencing the quality of AA surgical treatment (46). Although high SDI regions show a declining trend in AA disease burden, some of these regions still face challenges such as an aging population and will need to implement more targeted management measures in the future.

Our study also has some limitations. First, our analysis of disease burden is based on the GBD 2021 database, and the data from countries and regions with limited healthcare conditions may be underestimated. Second, our research primarily focused on mortality and DALYs, without analyzing other indicators such as incidence and prevalence of AA. Third, due to data limitations, our study did not conduct an analysis on the specific classifications of AA. Fourth, this study is a secondary analysis based on the GBD 2021 database and currently lacks cross-regional external validation cohorts. Future studies incorporating epidemiological data from more regions and countries are needed to validate our findings.

## Conclusion

In summary, AA represents a significant disease burden among individuals aged 60 and above globally, with a higher concentration in the male population. The burden of AA related to lead exposure shows notable regional disparities, highlighting the need for enhanced lead monitoring and control policies in high-risk areas. Over the next 15 years, AA-related mortality and DALYs rates are expected to decline globally; however, healthcare policies must be improved to address aging populations and SDI levels, identify high-risk areas and populations, and reduce the disease burden.

## Data Availability

The datasets presented in this study can be found in online repositories. The names of the repository/repositories and accession number(s) can be found at: https://vizhub.healthdata.org/gbd-results/.

## Footnotes

1
https://vizhub.healthdata.org/gbd-results/
