# Supplementary material for: Burden of aortic aneurysm and lead exposure risk factor in adults aged 60 years and older from 1990 to 2021: a global, regional, and national analysis
Source: Front Public Health. 2026 Apr 10;14:1696422. doi: 10.3389/fpubh.2026.1696422 (PMC13106329; doi:10.3389/fpubh.2026.1696422)
Supplement: Supplementary file 1 [file Data_Sheet_1.docx]

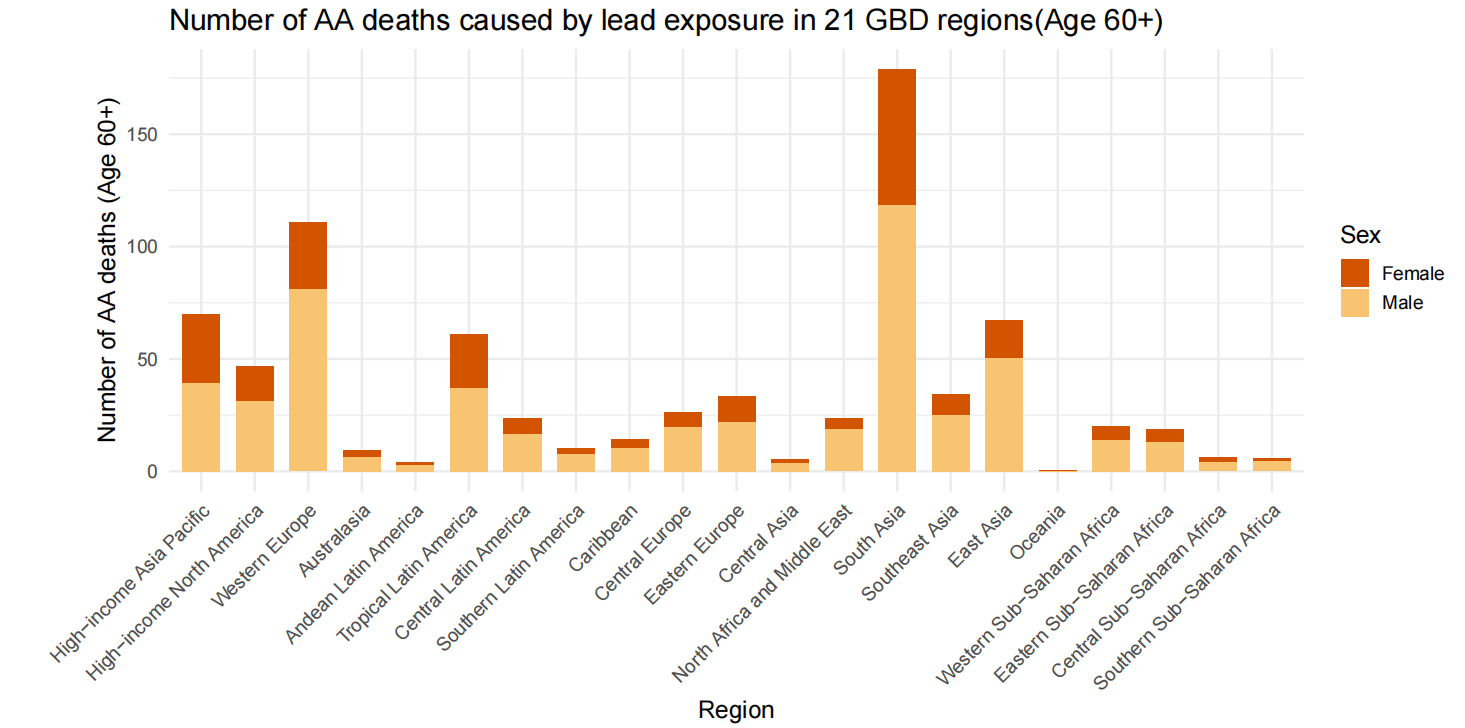


Figure S1 The Deaths Number of aortic aneurysm attributable to lead exposure among adults aged 60 years and older in the 21 GBD regions (per 100,000 population)


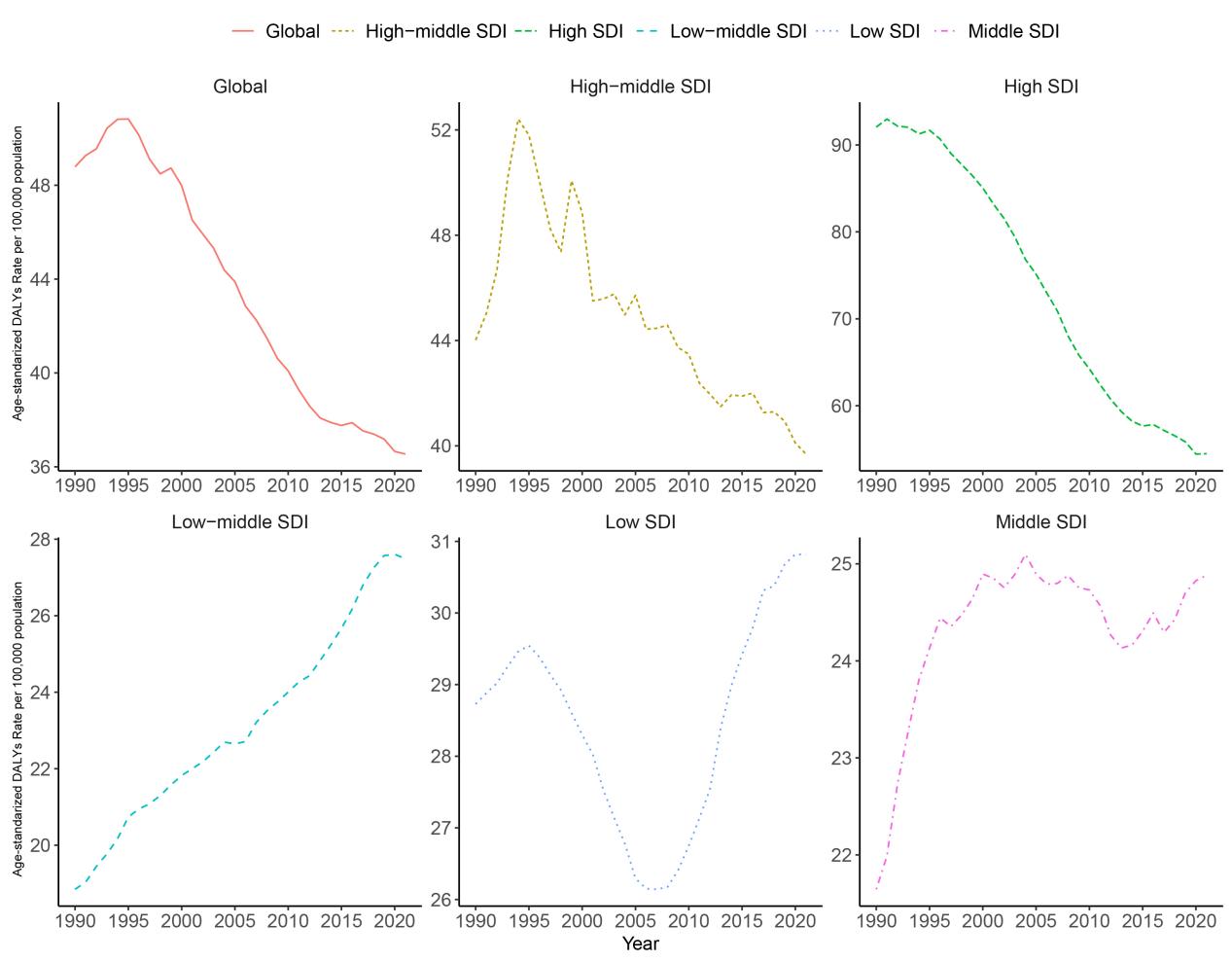


Figure S2 The trends in age-standardized DALYs rates from 1990 to 2021 globally and in 5 SDI regions.


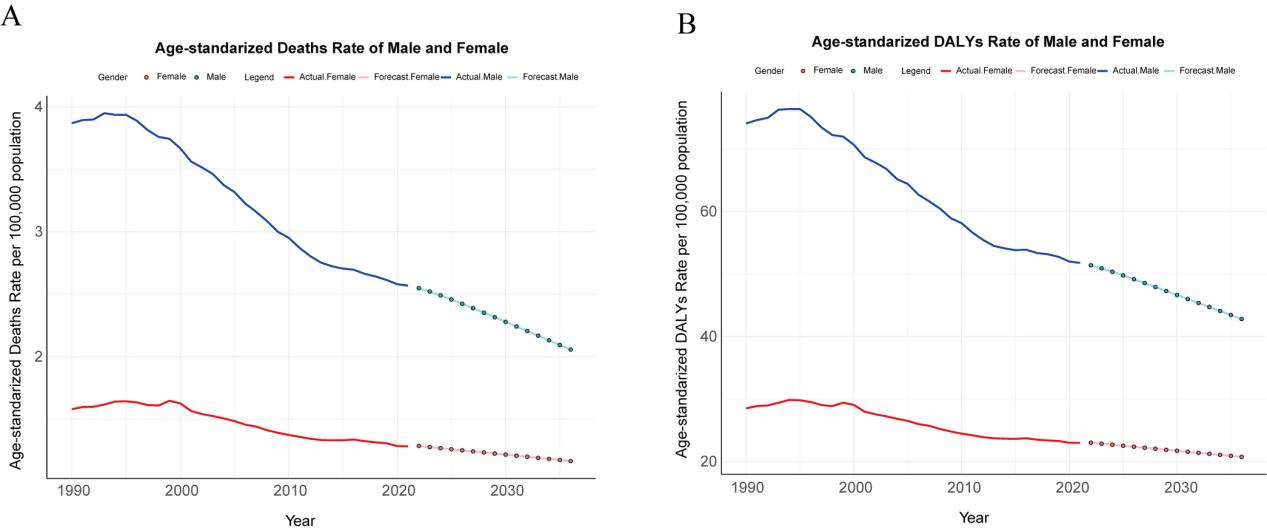


Figure S3 Using the ARIMA model to forecast disease trends for the next 15 years based on data from 1990 to 2021.(A) Forecast of age-standardized mortality rate for aortic aneurysm over the next 15 years; (B) Forecast of age-standardized DALYs rate for aortic aneurysm over the next 15 years;
