# Supplementary material for: Burden of aortic aneurysm and lead exposure risk factor in adults aged 60 years and older from 1990 to 2021: a global, regional, and national analysis
Source: Front Public Health. 2026 Apr 10;14:1696422. doi: 10.3389/fpubh.2026.1696422 (PMC13106329; doi:10.3389/fpubh.2026.1696422)
Supplement: Supplementary file 2 [file Table_1.docx]

| **Supplementary Table 1** Sex-specific differences in the numbers and rates of deaths and DALYs for aortic aneurysm attributable to lead exposure. | | | | | | | | |
| --- | --- | --- | --- | --- | --- | --- | --- | --- |
|  | **Male** | | | | **Female** | | | |
| **location_name** | **Number of cases, 1990** | **Rate of cases, 1990** | **Number of cases, 2021** | **Rate of cases, 2021** | **Number of cases, 1990** | **Rate of cases, 1990** | **Number of cases, 2021** | **Rate of cases, 2021** |
| Global | 558.006 (-75.563–1275.796) | 0.255 (-0.035–0.583) | 1156.492 (-130.676–2580.012) | 0.226 (-0.026–0.505) | 276.776 (-39.660–632.821) | 0.103 (-0.015–0.235) | 651.810 (-80.084–1430.573) | 0.110 (-0.014–0.243) |
| Andean Latin America | 1.208 (-0.123–3.195) | 0.105 (-0.011–0.278) | 9.059 (-0.759–22.966) | 0.251 (-0.021–0.637) | 0.755 (-0.080–2.029) | 0.061 (-0.006–0.163) | 4.481 (-0.390–11.959) | 0.111 (-0.010–0.296) |
| Australasia | 14.399 (-2.312–32.362) | 1.044 (-0.168–2.346) | 10.162 (-1.546–22.709) | 0.307 (-0.047–0.686) | 7.763 (-1.264–17.709) | 0.453 (-0.074–1.034) | 7.549 (-1.160–17.397) | 0.204 (-0.031–0.470) |
| Caribbean | 8.812 (-1.307–22.594) | 0.557 (-0.083–1.429) | 15.499 (-1.981–36.650) | 0.492 (-0.063–1.164) | 3.030 (-0.474–7.330) | 0.178 (-0.028–0.430) | 6.814 (-0.863–15.910) | 0.187 (-0.024–0.436) |
| Central Asia | 2.303 (-0.299–5.848) | 0.110 (-0.014–0.280) | 12.001 (-1.422–28.863) | 0.273 (-0.032–0.656) | 1.617 (-0.203–4.090) | 0.048 (-0.006–0.120) | 7.684 (-0.941–19.223) | 0.131 (-0.016–0.327) |
| Central Europe | 29.676 (-3.646–69.252) | 0.364 (-0.045–0.850) | 49.861 (-6.057–113.076) | 0.386 (-0.047–0.876) | 14.229 (-1.666–32.364) | 0.124 (-0.015–0.282) | 23.660 (-2.908–52.348) | 0.136 (-0.017–0.301) |
| Central Latin America | 10.503 (-1.503–24.496) | 0.228 (-0.033–0.532) | 33.500 (-4.216–78.934) | 0.234 (-0.029–0.552) | 4.134 (-0.583–9.553) | 0.082 (-0.012–0.189) | 15.755 (-1.947–36.399) | 0.093 (-0.011–0.214) |
| Central Sub-Saharan Africa | 3.167 (-0.575–9.892) | 0.266 (-0.048–0.830) | 14.297 (-2.372–43.514) | 0.527 (-0.087–1.605) | 1.489 (-0.330–5.613) | 0.109 (-0.024–0.411) | 6.548 (-0.890–21.472) | 0.185 (-0.025–0.607) |
| East Asia | 19.674 (-3.091–47.006) | 0.039 (-0.006–0.094) | 97.067 (-11.448–233.054) | 0.071 (-0.008–0.171) | 13.311 (-1.732–31.202) | 0.024 (-0.003–0.057) | 41.662 (-4.921–97.841) | 0.028 (-0.003–0.066) |
| Eastern Europe | 37.950 (-4.730–85.704) | 0.317 (-0.040–0.716) | 111.329 (-12.829–259.490) | 0.601 (-0.069–1.400) | 25.922 (-2.812–60.774) | 0.104 (-0.011–0.244) | 69.917 (-7.908–171.588) | 0.224 (-0.025–0.549) |
| Eastern Sub-Saharan Africa | 5.392 (-0.847–16.283) | 0.124 (-0.019–0.373) | 31.370 (-3.534–80.969) | 0.331 (-0.037–0.854) | 3.168 (-0.580–10.023) | 0.073 (-0.013–0.230) | 12.388 (-1.428–35.284) | 0.118 (-0.014–0.335) |
| High-income Asia Pacific | 28.892 (-3.332–65.033) | 0.270 (-0.031–0.608) | 96.486 (-9.941–221.518) | 0.374 (-0.039–0.858) | 16.300 (-1.813–35.691) | 0.111 (-0.012–0.243) | 96.705 (-10.887–229.747) | 0.306 (-0.034–0.726) |
| High-income North America | 90.563 (-12.028–211.116) | 0.466 (-0.062–1.086) | 41.363 (-5.387–96.978) | 0.103 (-0.013–0.242) | 46.413 (-5.877–111.546) | 0.174 (-0.022–0.418) | 28.557 (-3.436–66.006) | 0.061 (-0.007–0.141) |
| North Africa and Middle East | 5.454 (-0.705–16.232) | 0.055 (-0.007–0.164) | 32.593 (-4.305–80.805) | 0.124 (-0.016–0.308) | 2.388 (-0.311–6.257) | 0.025 (-0.003–0.065) | 12.548 (-1.739–30.169) | 0.047 (-0.006–0.113) |
| Oceania | 0.150 (-0.016–0.422) | 0.090 (-0.010–0.253) | 0.504 (-0.055–1.293) | 0.118 (-0.013–0.304) | 0.077 (-0.010–0.233) | 0.048 (-0.007–0.147) | 0.264 (-0.027–0.753) | 0.068 (-0.007–0.195) |
| South Asia | 26.909 (-3.556–86.728) | 0.080 (-0.011–0.258) | 204.818 (-24.091–613.529) | 0.236 (-0.028–0.708) | 20.367 (-3.630–58.410) | 0.066 (-0.012–0.189) | 120.214 (-15.438–282.097) | 0.133 (-0.017–0.311) |
| Southeast Asia | 11.187 (-1.574–31.848) | 0.083 (-0.012–0.237) | 57.697 (-6.114–140.019) | 0.156 (-0.017–0.379) | 6.138 (-0.973–17.207) | 0.039 (-0.006–0.109) | 25.938 (-2.972–61.374) | 0.059 (-0.007–0.139) |
| Southern Latin America | 19.397 (-2.357–51.063) | 0.752 (-0.091–1.980) | 21.394 (-2.854–54.319) | 0.428 (-0.057–1.086) | 6.195 (-0.727–15.961) | 0.186 (-0.022–0.478) | 10.678 (-1.482–25.276) | 0.165 (-0.023–0.390) |
| Southern Sub-Saharan Africa | 4.869 (-0.484–13.049) | 0.355 (-0.035–0.952) | 10.740 (-1.273–25.308) | 0.353 (-0.042–0.832) | 2.324 (-0.306–5.995) | 0.122 (-0.016–0.314) | 5.383 (-0.619–13.574) | 0.119 (-0.014–0.301) |
| Tropical Latin America | 16.612 (-2.158–38.721) | 0.332 (-0.043–0.773) | 54.876 (-5.903–124.221) | 0.381 (-0.041–0.863) | 7.555 (-0.968–17.941) | 0.130 (-0.017–0.309) | 37.289 (-4.538–85.177) | 0.208 (-0.025–0.474) |
| Western Europe | 206.466 (-26.270–458.730) | 0.660 (-0.084–1.467) | 186.647 (-22.207–434.637) | 0.349 (-0.041–0.812) | 84.784 (-10.411–188.131) | 0.189 (-0.023–0.419) | 91.775 (-11.096–216.318) | 0.143 (-0.017–0.336) |
| Western Sub-Saharan Africa | 14.425 (-2.017–43.694) | 0.293 (-0.041–0.887) | 65.228 (-8.345–182.584) | 0.630 (-0.081–1.765) | 8.814 (-1.677–27.276) | 0.173 (-0.033–0.534) | 26.002 (-3.284–71.411) | 0.227 (-0.029–0.623) |
